# Disrupted rich club organization in structural brain networks is related to childhood maltreatment in major depressive disorder

**DOI:** 10.3389/fpsyt.2026.1759133

**Published:** 2026-02-26

**Authors:** Junhua Huang, Bei Rong, Guoqing Gao, Mingzhe Zhou, Haomian Zhao, Yu Gao, Ning Tu, Lihong Bu, Ling Xiao, Gaohua Wang

**Affiliations:** 1 Department of Psychiatry, Renmin Hospital of Wuhan University, Wuhan, Hubei, China; 2 Institute of Neuropsychiatry, Renmin Hospital of Wuhan University, Wuhan, Hubei, China; 3 Department of Psychiatry, Affiliated Psychological Hospital of Anhui Medical University, Hefei, China; 4 Department of Psychiatry, Hefei Fourth People’s Hospital, Hefei, China; 5 Department of Psychiatry, Anhui Mental Health Center, Hefei, China; 6 PET-CT/MR Center, Renmin Hospital of Wuhan University, Wuhan, Hubei, China; 7 Taikang Center for Life and Medical Sciences, Wuhan University, Wuhan, Hubei, China

**Keywords:** childhood maltreatment, connections, major depressive disorder, rich club organization, structural brain networks

## Abstract

**Background:**

Childhood maltreatment plays an important role for developing major depressive disorder (MDD), and many studies have suggested brain structural alterations related to this psychological factor. However, the specific impact of childhood maltreatment on rich club organization in structural brain networks in MDD remains unclear. The aim of this study was to investigate whether childhood maltreatment was related to the disruption of rich club organization in structural networks in MDD.

**Methods:**

In this cross-sectional study, we recruited 130 first-episode, drug-naïve MDD patients and 122 healthy controls (HCs). The structural brain networks were reconstructed for all participants based on diffusion imaging data. Subsequently, the rich club organization was determined, and the connectivity measures (strength and density) in different connection classes were calculated and compared. The relationships between connectivity measures and clinical scores were evaluated.

**Results:**

The MDD patients with childhood maltreatment showed significant decreased connectivity strength and density in rich club connections, as well as increased connectivity density in feeder connections, as compared to those patients without childhood maltreatment. Besides, HCs with childhood maltreatment had lower connectivity strength and density in feeder connections than that of HCs without childhood maltreatment. Moreover, the correlations between scores of childhood maltreatment and connectivity density in feeder connections were significantly positive in MDD, whereas these correlations exhibited negative in HCs.

**Conclusion:**

Our results may reflect the associations between the disrupted rich club organization and childhood maltreatment in MDD. Furthermore, with exposure to childhood maltreatment, the distinct connection patterns in depressed and healthy populations may indicate neuroimaging features associated with individual vulnerability or resilience to developing MDD in context of early life stress.

## Background

Major depressive disorder (MDD) is a common mental disorder, and has become a leading cause of disease burden worldwide (1). Despite its destructive implications, the neurobiological mechanisms underlying MDD are not fully elucidated, thus limiting the early detection and intervention of this disorder. In recent years, childhood maltreatment has been identified as a major risk factor for MDD. A meta-analysis showed that approximately 46% of individuals with depression reported childhood maltreatment (2). In a more recent study, 55.5% of patients with depression reported at least one type of maltreatment in childhood (3). Furthermore, previous findings indicated that the experiences of childhood maltreatment were linked to chronic disease trajectory and poorer treatment outcomes in depression (2, 4). Therefore, childhood maltreatment is highly associated with the development of depression, and investigating the neurobiological mechanisms linking childhood maltreatment and depression may provide insights into neuropathology underlying this disorder, thus further facilitating early and effective interventions.

Emerging evidence from neuroimaging studies have suggested that childhood maltreatment is associated with brain structural changes in MDD. Several morphological studies based on region of interest (ROI) analysis have revealed that volumes in hippocampus, amygdala and anterior cingulate cortex were decreased in healthy individuals with childhood maltreatment (5–7). Another neuroimaging study based on ROI analysis and machine learning also demonstrated that young adults with childhood maltreatment exposure had reduced surface areas and cortical thicknesses in extensive fronto-temporal regions (8). In addition to morphological alterations in brain structure, recent studies have revealed the associations between structural connectivity and childhood maltreatment. Based on tract-based spatial statistics (TBSS) analysis, a prior diffusion tensor imaging (DTI) study reported shorter fiber bundles passed through the right posterior corona radiata, right anterior corona radiata, and anterior thalamic radiation in healthy adults with childhood maltreatment compared with those without childhood maltreatment (9). In addition, a meta-analysis including whole-brain voxel-based DTI studies showed that individuals with childhood maltreatment had significantly reduced fractional anisotropy (FA) in the left anterior thalamic radiation and bilateral fornix, optic radiations, inferior longitudinal fasciculus, and inferior frontal-occipital fasciculus, along with the anterior portions of the corpus callosum (10). Paralleling the effects of childhood maltreatment, the patients with depression showed similar alterations in white matter microstructure. According to a meta-analysis including DTI studies with TBSS or voxel-based analysis, the patients with MDD showed decreased FA in white matter regions that mainly involved the fascicles of right inferior longitudinal fasciculus, right inferior fronto-occipital fasciculus, right posterior thalamic radiation and interhemispheric fibers running through the genu and body of the corpus callosum (11). A more recent study using TBSS method demonstrated that first-episode, treatment-naïve adolescents with MDD had reduced FA in cingulum, forceps minor, inferior fronto-occipital fasciculus, inferior longitudinal fasciculus, superior longitudinal fasciculus, and uncinate fasciculus (12). The overlap in white matter changes associated with depression and childhood maltreatment indicates that brain alterations in structural connection could play an important role in the pathological link between childhood maltreatment and depression. As such, research on brain structural connectivity and its putative role for childhood maltreatment in MDD has received increased attention in recent years. In a DTI study using TBSS analysis, Kang et al. found that childhood maltreatment was negatively correlated with the FA in corpus callosum and crossing pontine tract in MDD patients (13). Similarly, another study based on TBSS analysis discovered that in MDD patients, adverse childhood experiences were negatively correlated with white matter integrity in superior and inferior longitudinal fasciculus, inferior fronto-occipital fasciculus, corpus callosum, cingulate gyrus, forceps major and minor, thalamic radiation, and internal capsule (14). These findings indicate that childhood maltreatment is linked to the disruption of widespread structural connectivity in brain, which have advanced our understanding of the neurobiology basis bridging childhood maltreatment and MDD.

However, beyond impairment in structural connectivity, there is still a gap in understanding how the brain is influenced by childhood maltreatment at a network level in the development of MDD. In recent years, graph theory has been a powerful framework for exploring the topological properties in brain connectivity network, such as the small-worldness, network efficiency, and rich club organization. Currently, there are only preliminary findings regarding the effect of childhood maltreatment on topological alterations in structural connectome. In a study including healthy adults, subjects with moderate-to-high exposure to maltreatment showed lower global efficiency and higher small-worldness in structural networks (15). However, another study with a large transdiagnostic sample showed contradictory results. And in this study, individuals with a history of childhood maltreatment had increased network connections and efficiency. Linda et al. indicated that these findings might reflect a hyperconnectivity pattern as compensatory response to early adversity (16). Similar to the effect of childhood treatment, reduced efficiency in structural networks were reported in patients with MDD (17, 18). Moreover, there was a reduction of rich club connections in adolescent MDD and late-life depression patients compared with healthy controls (19, 20). The rich club organization supports segregated and integrated information processing, thus playing a critical role in maintaining the global communication in the most efficient way in human brain connectome (21, 22). As such, alterations in rich club organization could be an important characteristic for brain network dysfunction in MDD. Considering that depression is characterized by the disruption of rich club connections, and childhood maltreatment is a potent risk factor known to comprise white matter alterations, it is logical to nourish the interpretation that disturbance of rich club organization could be associated with childhood maltreatment in depression. And this hypothesis could be supported by previous literatures. Chronic stress is associated with hypothalamic-pituitary-adrenal (HPA) axis dysregulation and neuroinflammation, and then activated glial cells could release reactive oxygen and nitrogen species (23). As rich club regions are presented with a continuously high baseline activity and glucose metabolism, they could be particularly vulnerable to harmful mechanism such as neuroinflammation and oxidative stress (24). However, the neuroimaging studies *in vivo* that demonstrate the link between disruption of rich club organization and childhood maltreatment in MDD are still lacking.

In this study, we used diffusion imaging data and graph theory together to investigate possible associations between childhood maltreatment and the disruption of rich club organization in structural brain networks in first-episode, drug-naïve MDD. On the basis of previous findings, we hypothesized that in MDD the structural network would show disrupted rich club organization, and these alterations might be linked to childhood maltreatment.

## Materials and method

### Participants

This study was approved by the Clinical Research Ethic Committee, Renmin Hospital of Wuhan University, and was conducted under the 1964 Helsinki Declaration and its later amendments or comparable ethical standards. Each participant received a full explanation of this study and provided written informed consent before the study. A total of 130 patients with first-episode, drug-naïve MDD were recruited from Department of Psychiatry, Renmin Hospital of Wuhan University, Wuhan, China. All patients were right-handed and aged over 18 years. The diagnosis of MDD was determined by two experienced clinical psychiatrists according to the criteria of the Diagnostic and Statistical Manual of Mental Disorders, Fifth Edition (DSM-V) in the Structured Clinical Interview. Patients were excluded if they had (1) any history of neurological disorder or head trauma; (2) history of other psychiatric disorders; (3) alcohol/substance abuse or dependence; and (4) the presence of any concurrent sever physical disease. Meanwhile, a total of 122 right-handed healthy controls (HCs) matched for sex, age and education level were recruited through local media advertisements in Wuhan, China. None of HCs had the presence of current or past psychiatric diagnosis. Furthermore, the HCs had no current or past significant neurological illness, substance abuse or dependence, or any history of psychiatric illness in first-degree relatives.

The clinical characteristics of patients were assessed using the 24-item Hamilton Depression Rating Scale (HAMD) and Hamilton Anxiety Rating Scale (HAMA). The childhood maltreatment experiences were assessed through the 28-item Childhood Trauma Questionnaire (CTQ) (25). This retrospective and self-reported questionnaire contains five subscales, including emotional, physical and sexual abuse, and emotional and physical neglect. The cut-off scores of these subscales are as follows: emotional abuse ≥ 13, physical abuse ≥ 10, sexual abuse ≥ 8, emotional neglect ≥ 15, physical neglect ≥ 10 (26, 27). The individuals who exceeded the cut-off threshold for at least one subscale were considered to experience childhood maltreatment, whereas those having scores below the cut-off threshold for all five subscales were assigned into non-childhood maltreatment group. Therefore, the participants were divided into four groups: MDD patients with childhood maltreatment (MDD-CM), MDD patients without childhood maltreatment (MDD-nCM), HCs with childhood maltreatment (HC-CM), and HCs without childhood maltreatment (HC-nCM).

### Imaging data acquisition

All magnetic resonance imaging (MRI) data were acquired on a 3.0 T MRI scanner (Discovery MR750w, GE Healthcare, Milwaukee, WI) at PET-CT/MR Center, Renmin Hospital of Wuhan University, Wuhan, China. The diffusion imaging data of brain were obtained using a diffusion-weighted spin-echo Echo Planar Imaging (EPI) sequence with two b values (b = 1000 and 2000 s/mm^2^) across a total of 60 gradient directions, supplemented by two non-diffusion weighted *b_0_* volumes. Other diffusion imaging parameters were as follows: repetition time (TR) = 15000 ms; echo time (TE) = 88 ms; flip angle = 90°; slice thickness = 2.0 mm; slice gap = 0.0 mm; field of view (FOV) = 256 mm × 256 mm; matrix = 128 × 128; voxel size = 2.0 mm × 2.0 mm × 2.0 mm; 73 slices. T1-weighted anatomical images were acquired through the BRAVO sequence with the following parameters: TR = 8.5 ms; TE = 3.2 ms; flip angle = 12°; slice thickness = 1.0 mm; gap = 0.0 mm; FOV = 256 mm × 256 mm; matrix = 256 × 256; voxel size = 1.0 mm × 1.0 mm × 1.0 mm; 176 slices.

### Data processing

Diffusion MRI data were preprocessed using mritrix3 (https://www.mrtrix.org/) and FMRIB Software Library (FSL) (https://fsl.fmrib.ox.ac.uk/fsl/). The diffusion and T1-weighted data were converted from DICOM to NIFTI format. And then skull stripping was conducted for these imaging data. The subsequent processing steps for diffusion images included principal component analysis (PCA) denoising, Gibbs ringing artifacts removal, and eddy current and head motion correction. Then we estimated voxel-wise diffusion tensor and obtained the FA maps. Subsequently, the whole brain deterministic tractography was performed for each participant in native diffusion space using the Fiber Assignment by Continuous Tracking (FACT) algorithm embedded in the Diffusion Toolkit. Fiber tracking was terminated either the FA < 0.2 or the angle between the current and the previous path segment exceeded 45° (28, 29).

### Network construction

In this study, we used Automated Anatomical Labeling (AAL) atlas to parcellate the whole brain, and then 90 cortical and subcortical regions were included as regions of interest (ROIs). To construct structural network individually, the ROIs were defined in the native diffusion space. Specifically, the *b_0_* maps in native diffusion space were coregistered to their corresponding T1-weighted images. Subsequently, individual T1-weighted images were nonlinearly registered to the Montreal Neurological Institute (MNI) space. The transformations derived from these two steps were combined and inversed, and then the transformation parameters were used to warp the AAL atlas mask from the MNI space to the individual native diffusion space. For a brain structural network, the nodes were determined as 90 ROIs, and the fiber numbers connecting between each pair of brain ROIs were calculated and defined as the edges. As a result, a 90 × 90 matrix were constructed for each participant. To exclude the spurious edges that resulted from the potential effect of noise or other factors during data acquisition and processing, the structural connections with at least 3 fiber numbers were considered to exist (30, 31).

### Rich club organization

In a network, the degree of a specific node is defined as the total number of edges that link directly to it. Within the network with characteristic of rich club organization, the nodes with high degree tend to be more strongly interconnected than expected based on their high degree alone. The presence of rich club characteristics could be analyzed through several measures according to previous literatures (32–34). In brief, the degree of each node in a network was calculated, and the parameter *k* was defined as the number of edges attached to a network node. As such, the *k* ranged from minimum degree to maximum degree in the network. For each *k* value in this range, the nodes that showed degree lower that *k* were removed from the network. In the network with remaining nodes and all edges among them, the rich club coefficient *Φ* was defined as the ratio of existing connections and the possible maximum connections of the network. Then the rich club coefficient was typically normalized to a set of random networks. In the present study, 1,000 random networks with the same size were constructed, and the rich club coefficient of each random network was calculated. The random rich club coefficient *Φ_random_* was computed as the average rich club coefficient over the 1,000 random networks. Finally, the normalized rich club coefficient *Φ_norm_* was computed as the ratio of *Φ* to *Φ_random_*. The rich club architecture in a network could be verified by a *Φ_norm_* greater than 1 across a range of *k* values. In this study, the calculation of degree and rich club coefficients were performed using a graph theoretical network analysis toolbox GRETNA (http://www.nitrc.org/projects/gretna/) based on MATLAB environment.

To define the rich club regions, we constructed a group-averaged structural network for each group. The group-averaged network was computed by selecting connections that were present in at least 60% subjects of the group (19, 35). The degree of each node was calculated based on the group-averaged network, and the top 12% nodes with highest degree were defined as rich club regions for this group (36, 37). Subsequently, the connections of individual brain network were divided into three classes: (1) rich club connections which link rich club regions each other, (2) feeder connections which link rich club regions to non-rich club regions, and (3) local connections which link non-rich club regions each other. Based on these connection classes, two measures of connectivity were calculated and compared. The connectivity strength was defined as the sum of all edge weights within a connection class, whereas the connectivity density was the ratio of connectivity strength of a connection class to that of the whole brain network (38).

### Statistical analysis

Statistical analyses of demographic and clinical data were performed using Rstudio (version 2024.04.1 + 748), under R version 4.4.0. The chi-squared test was used to determine the sex difference among MDD-CM, MDD-nCM, HC-CM, and HC-nCM groups. The differences in age, years of education, total scores and subscale scores of CTQ among these groups were compared with analysis of variance (ANOVA). For the variables with significant differences in ANOVA, the *post-hoc* tests were conducted using Tukey’s honestly significant difference (HSD) method. The total scores of HAMD and HAMA in MDD patients were compared using the two-sample t-tests. The statistical significance was set at *P* < 0.05.

The differences in connectivity strength and density, as well as nodal degree in rich club regions, among MDD-CM, MDD-nCM, HC-CM, and HC-nCM group were determined using non-parametric permutation tests (10,000 times). The *P* values in permutation tests were corrected for multiple comparisons using false discovery rate (FDR) method. Partial correlation analysis was used to assess the relationships between connectivity measures and total scores and subscale scores of CTQ within MDD and HCs groups separately. Additionally, the correlation between connectivity measures and total scores of HAMD and HAMA was assessed in MDD patients. For correlation analysis, we took age, sex, and education levels as nuisance covariates and regressed them out.

## Results

### Demographic and clinical characteristics

There were no significant differences in sex, age and years of education among MDD-CM, MDD-nCM, HC-CM, and HC-nCM groups. However, the patients with childhood maltreatment displayed higher HAMD and HAMA scores compared to those patients without childhood maltreatment. Additionally, these four groups showed significant differences in CTQ scores and subscale scores. Specifically, MDD-CM group had higher CTQ scores and all subscale scores than those of MDD-nCM group. Besides, compared with HC-CM group, the MDD-CM group showed increased CTQ scores and subscale scores of EA, EN and PN. Additionally, the MDD-CM group had elevated CTQ scores and all subscale scores than that of HC-nCM group. Compared with HC-CM group, MDD-nCM group had lower scores in PN. Also, the MDD-nCM group showed higher CTQ scores, as well as subscale scores in EA and EN, than that of HC-nCM group. Moreover, compared with HC-nCM group, the HC-CM group had higher CTQ scores and subscale scores in EA, PA, EN, and PN. Detailed demographic and clinical characteristics of all participants were shown in **Table 1**, **Supplementary Table S1**.

**Table 1:** Demographic and clinical characteristics of the participants.

Variable	MDD-CM	MDD-nCM	HC-CM	HC-nCM	Statistical value	*P* value
Sex (F/M)	67/17	28/18	21/11	58/32	7.019	0.071^a^
Age (years)	25.45 ± 5.61	26.76 ± 8.00	26.59 ± 7.26	27.12 ± 7.65	0.867	0.459^b^
Education (years)	14.83 ± 2.24	15.00 ± 2.70	15.47 ± 2.59	15.31 ± 2.26	0.865	0.460^b^
HAMD	31.92 ± 9.41	27.67 ± 9.87	–	–	2.417	0.017^c*^
HAMA	21.45 ± 7.77	18.11 ± 7.43	–	–	2.382	0.019^c^
CTQ	55.25 ± 13.46	36.98 ± 6.61	41.81 ± 8.84	30.38 ± 4.06	110.614	< 0.001^b*^
EA	11.57 ± 4.69	7.65 ± 1.99	8.25 ± 3.20	5.92 ± 1.40	47.090	< 0.001^b*^
PA	7.81 ± 3.68	5.54 ± 0.81	6.78 ± 2.94	5.29 ± 0.55	18.042	< 0.001^b*^
SA	6.68 ± 3.20	5.09 ± 0.35	5.94 ± 2.42	5.10 ± 0.37	10.350	< 0.001^b*^
EN	16.69 ± 4.19	10.41 ± 2.48	11.41 ± 4.83	8.06 ± 2.55	91.680	< 0.001^b*^
PN	11.45 ± 3.64	7.11 ± 1.62	9.44 ± 2.64	6.01 ± 1.26	73.956	< 0.001^b*^

Numbers are reported for sex data. All quantitative data are expressed as mean ± standard deviation.

MDD-CM, major depressive disorder with childhood maltreatment; MDD-nCM, major depressive disorder without childhood maltreatment; HC-CM, healthy controls with childhood maltreatment; HC-nCM, healthy controls without childhood maltreatment; F, female; M, male; HAMD, 24-item Hamilton Depression Rating Scale; HAMA, Hamilton Anxiety Rating Scale; CTQ, Childhood Trauma Questionnaire; EA, emotional abuse; PA, physical abuse; SA, sexual abuse; EN, emotional neglect; PN, physical neglect.

a *P* value obtained using the chi-squared test.

b *P* value obtained using the analysis of variance.

c *P* value obtained using the two-tailed two sample *t* test.

*P value < 0.05, statistically significant.

### Rich club organization

**Figure 1:**
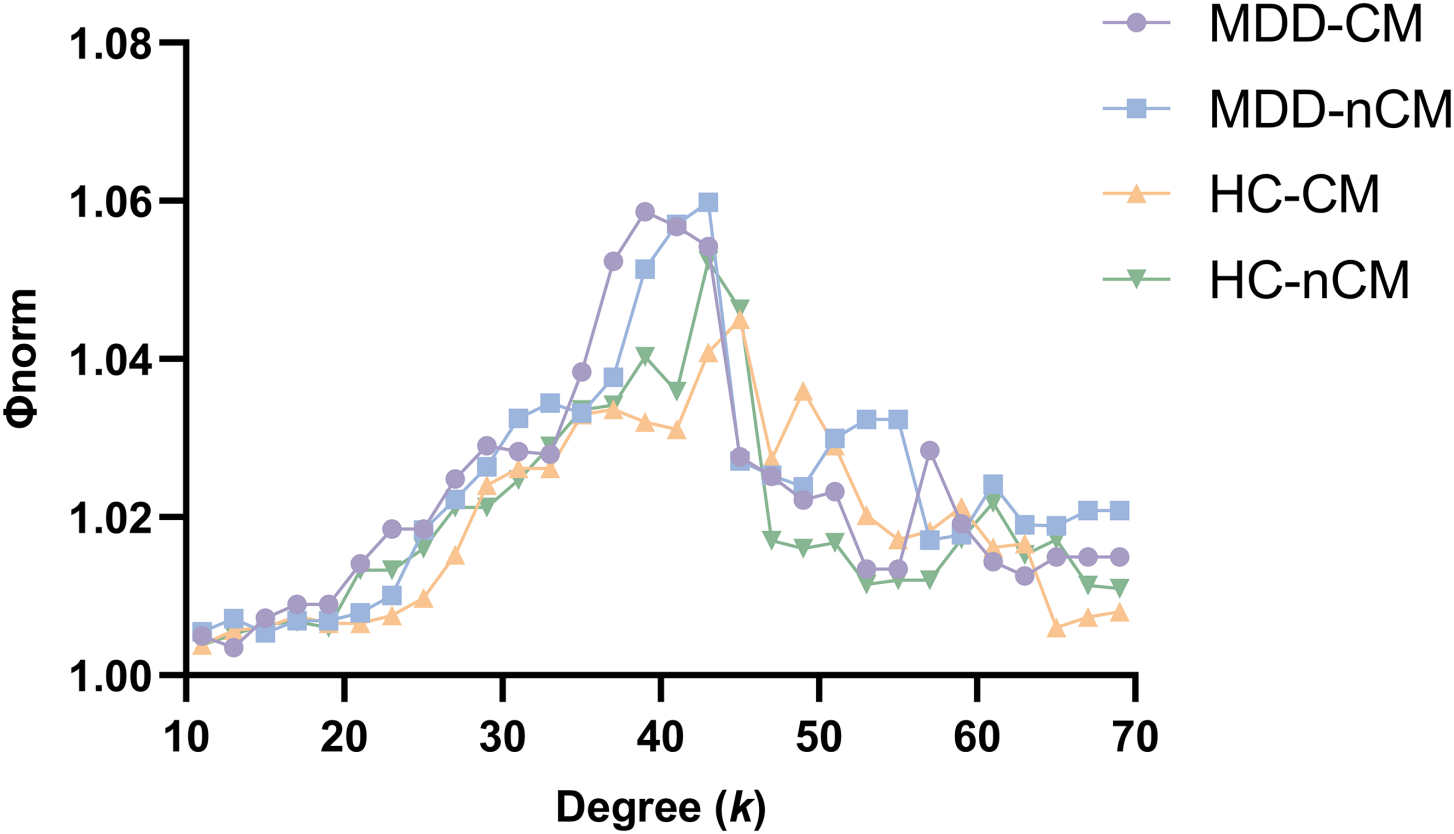
Group-averaged rich club curves for MDD-CM, MDD-nCM, HC-CM and HC-nCM groups. Φ_norm_, normalized rich club coefficient; MDD-CM, major depressive disorder with childhood maltreatment; MDD-nCM, major depressive disorder without childhood maltreatment; HC-CM, healthy controls with childhood maltreatment; HC-nCM, healthy controls without childhood maltreatment.

**Figure 2:**
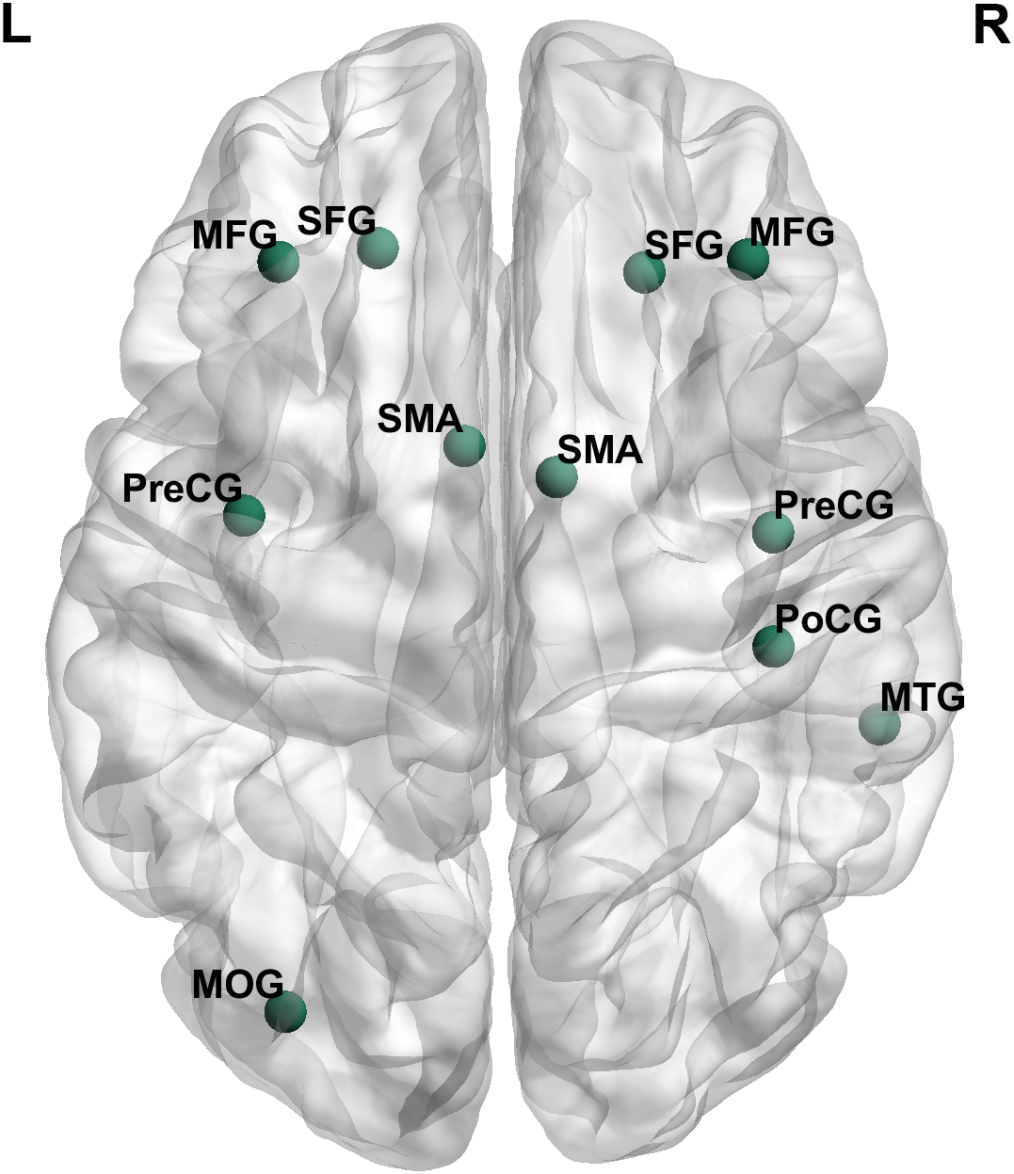
Rich club regions identified in MDD-CM, MDD-nCM, HC-CM, and HC-nCM groups. L, left; R, right; MFG, middle frontal gyrus; SFG, superior frontal gyrus; PreCG, precentral gyrus; SMA, supplementary motor area; PoCG, postcentral gyrus; MTG, middle temporal gyrus; MOG, middle occipital gyrus; MDD-CM, major depressive disorder with childhood maltreatment; MDD-nCM, major depressive disorder without childhood maltreatment; HC-CM, healthy controls with childhood maltreatment; HC-nCM, healthy controls without childhood maltreatment.

The presence of rich club organization in structural brain networks for each group was verified, as denoted by group-averaged *Φ_norm_* > 1 over a range of degree (*k*) (**Figure 1**). Subsequently, we found that the rich club regions identified in all groups shared a common set of nodes, which included the bilateral middle frontal gyrus (MFG), bilateral superior frontal gyrus (SFG), bilateral precentral gyrus (PreCG), bilateral supplementary motor areas (SMA), left middle occipital gyrus (MOG), right postcentral gyrus (PoCG), and right middle temporal gyrus (MTG) (**Figure 2**).

### Connectivity measures of rich club, feeder and local connections

**Figure 3:**
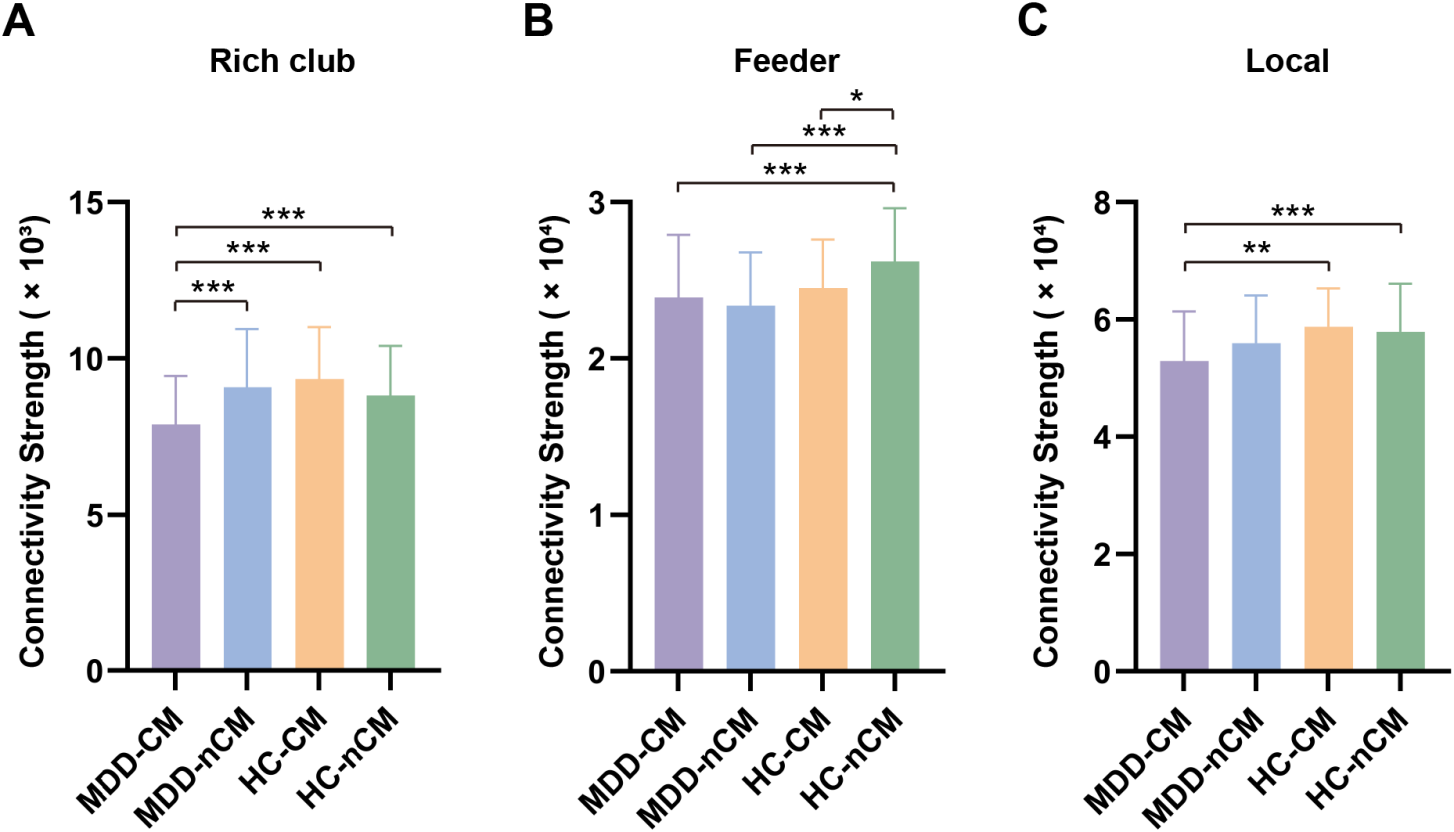
Group differences of connectivity strength in rich club **(A)**, feeder **(B)**, and local **(C)** connections among MDD-CM, MDD-nCM, HC-CM, and HC-nCM groups. MDD-CM, major depressive disorder with childhood maltreatment; MDD-nCM, major depressive disorder without childhood maltreatment; HC-CM, healthy controls with childhood maltreatment; HC-nCM, healthy controls without childhood maltreatment. *, corrected *P* value < 0.05; **, corrected *P* value < 0.01; ***, corrected *P* value < 0.001.

Based on the categorization of rich club and non-rich club regions in 90 nodes, the edges of structural networks were classified into rich club, feeder and local connections. There were significant differences in the connectivity strength of these connections among MDD-CM, MDD-nCM, HC-CM and HC-nCM groups. Specifically, the MDD-CM group showed decreased connectivity strength in rich club connections than that of MDD-nCM group. Additionally, the MDD-CM group displayed significantly lower connectivity strength in rich club and local connections compared to HC-CM group. Besides, the connectivity strength of all three connection classes was significantly reduced in MDD-CM group as compared with HC-nCM group. Relative to HC-nCM group, the MDD-nCM and HC-CM groups also showed decreased connectivity strength in feeder connections. (**Figure 3**; **Supplementary Table S2**).

**Figure 4:**
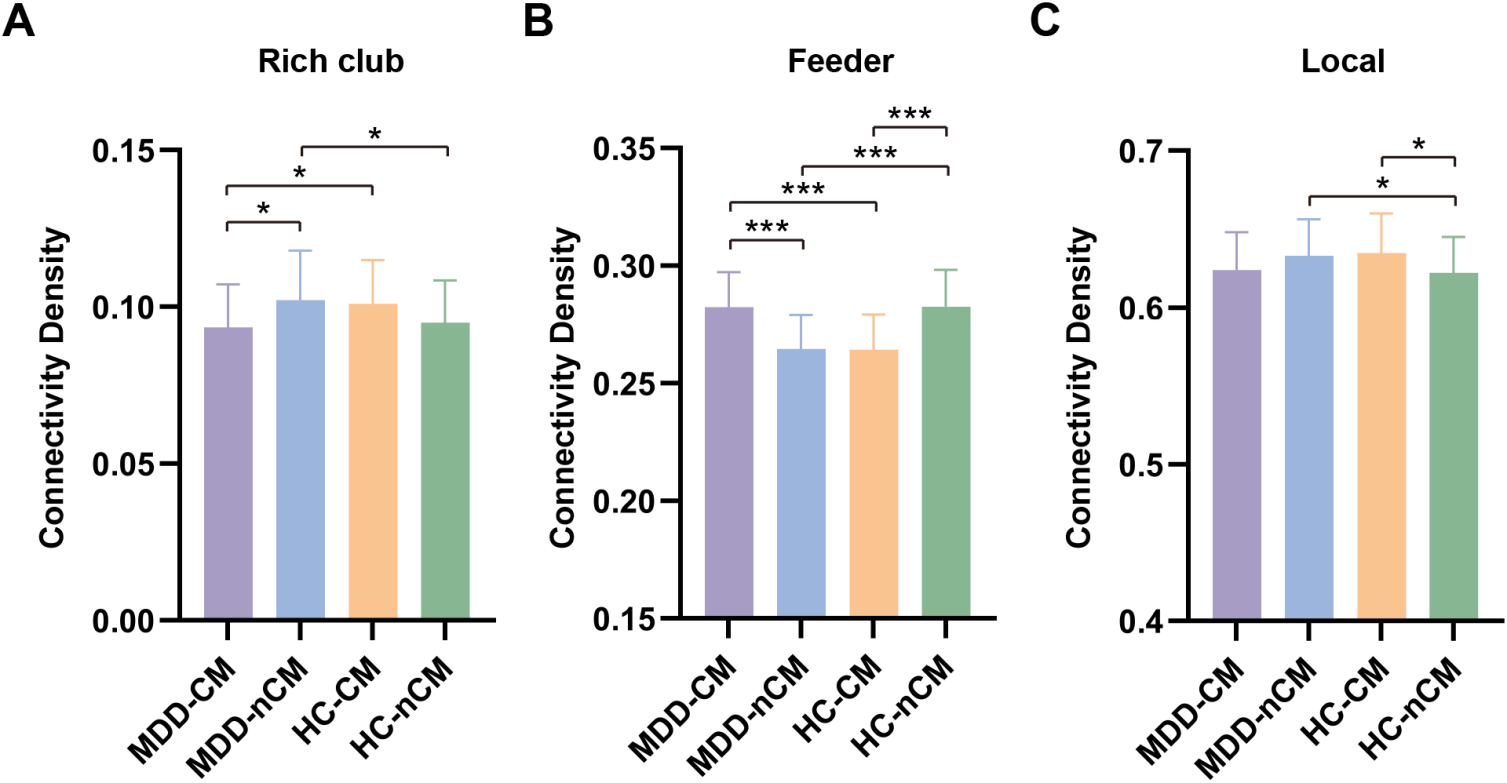
Group differences of connectivity density in rich club **(A)**, feeder **(B)**, and local **(C)** connections among MDD-CM, MDD-nCM, HC-CM, and HC-nCM groups. MDD-CM, major depressive disorder with childhood maltreatment; MDD-nCM, major depressive disorder without childhood maltreatment; HC-CM, healthy controls with childhood maltreatment; HC-nCM, healthy controls without childhood maltreatment. *, corrected *P* value < 0.05; ***, corrected *P* value < 0.001.

Our study also found significant connectivity density differences among groups in rich club, feeder and local connections. The MDD-CM group exhibited lower connectivity density in rich club connections, as well as greater connectivity density in feeder connections than that of MDD-nCM and HC-CM groups. Besides, the MDD-nCM group showed increased connectivity density in rich club and local connections, as well as decreased connectivity density in feeder connections, as compared with HC-nCM groups. In addition, the HC-CM group displayed reduced connectivity density in feeder connections, as well as elevated connectivity density in local connections, as compared to HC-nCM group. (**Figure 4**; **Supplementary Table S3**).

### Nodal degree in rich club regions

In this study, there were also significant differences in nodal degree in several rich club regions among these groups. Specifically, compared with HC-nCM group, the MDD-CM group showed decreased nodal degree in bilateral SFG, right PreCG, right MTG, and right MFG. Additionally, MDD-nCM had lower nodal degree in right MTG and MFG than that of HC-nCM group. Moreover, compared with HC-nCM group, HC-CM group exhibited a reduction of nodal degree in right MFG. (**Supplementary Table S4**).

### Correlations with clinical characteristics

**Figure 5:**
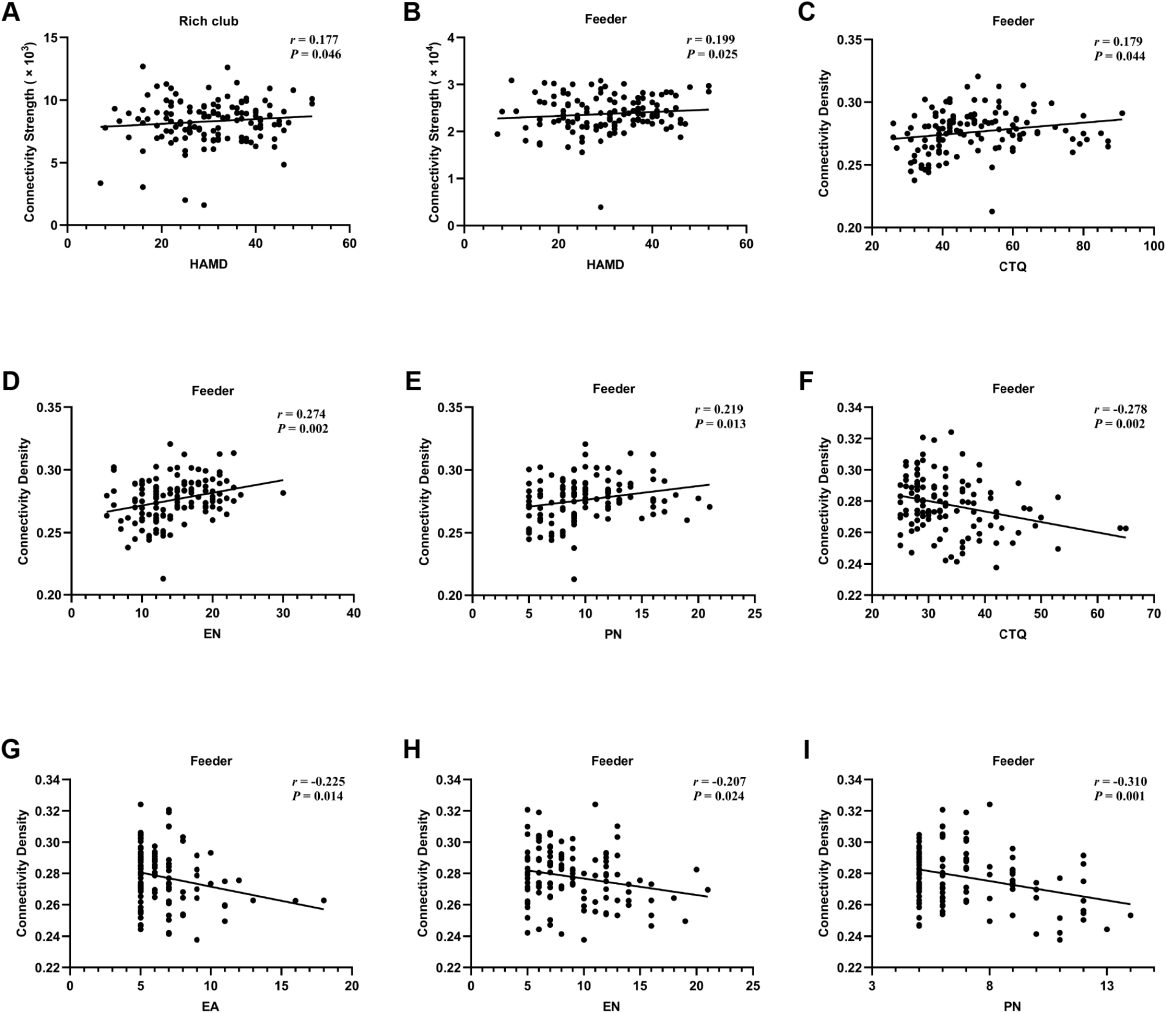
Correlations between connectivity measures and clinical data in MDD and HCs. **(A)** Correlation between connectivity strength in rich club connections and total scores of HAMD in MDD. **(B)** Correlation between connectivity strength in feeder connections and total scores of HAMD in MDD. **(C-E)** Correlations between connectivity density in feeder connections and total scores, EN scores, and PN scores of CTQ in MDD. **(F-I)** Correlations between connectivity density in feeder connections and total scores, EA scores, EN scores, and PN scores of CTQ. MDD, major depressive disorder; HCs, healthy controls; HAMD, 24-item Hamilton Depression Rating Scale; CTQ, Childhood Trauma Questionnaire; EN, emotional neglect; PN, physical neglect; EA, emotional abuse.

The results of correlation analysis showed that connectivity strength in rich club and feeder connections was positively correlated with the total scores of HAMD in patients with MDD (*r* = 0.177, *P* = 0.046; *r* = 0.199, *P* = 0.025, respectively). Additionally, there were positive correlations between the connectivity density in feeder connections and the total scores, EN scores, and PN scores of CTQ in patients with MDD (*r* = 0.179, *P* = 0.044; *r* = 0.274, *P* = 0.002; *r* = 0.219, *P* = 0.013, respectively). However, the connectivity density in feeder connections exhibited negative correlations with the total scores, EA scores, EN scores, and PN scores of CTQ in HCs (*r* = -0.278, *P* = 0.002; *r* = -0.225, *P* = 0.014; *r* = -0.207, *P* = 0.024; *r* = -0.310, *P* = 0.001, respectively) (**Figure 5**).

## Discussion

In the current study, we identified the disruption of rich club organization in structural brain networks in first-episode, drug-naïve MDD, as well as their links to childhood maltreatment. Specifically, we found that MDD-CM group had reduced connectivity strength and density in rich club connections, as well as elevated connectivity density in feeder connections, as compared with MDD-nCM group. In addition, HC-CM group showed lower connectivity strength and density in feeder connections as compared with HC-nCM group. Moreover, there were positive correlations between HAMD scores and connectivity strength in rich club and feeder connections in patients with MDD. Furthermore, there were positive correlations between CTQ scores and connectivity density in feeder connections in MDD, whereas these correlations showed negative in HCs. These findings may extend our understanding of neuropathological mechanism underlying the associations between childhood maltreatment and MDD from a perspective of structural connectivity connectome.

The rich club organization is considered to play a critical role in the information integration among segregated communities in brain, and the abnormalities of rich club organization in brain networks has been demonstrated to be associated with depression (21). Liu et al. revealed that the rich club and feeder connections were significantly decreased in MDD patients (39). Similarly, reduced connectivity strength of rich club, feeder and local connections were reported in first-episode, drug-naïve adolescent MDD (19). Moreover, with the remission of depressive symptoms, MDD patients exhibited enhanced rich club connectivity in functional brain networks (40). These findings indicated that there was a widespread dysconnectivity pattern with disrupted integration of neural information underlying MDD. In this study, we further discovered that the MDD-CM group showed lower connectivity strength and density in rich club connections compared to MDD-nCM group. Our results demonstrated the association between childhood maltreatment and the dysconnectivity pattern among rich club regions in depression. Interestingly, the MDD-CM group also exhibited significantly increased connectivity density in feeder connections. One possible interpretation is that the increased connectivity density of feeder connections could be a secondary result of the reduced proportion of rich club connection. As rich club connections showed a reduction in connectivity strength and density, the non-hub connections could mathematically manifest a concomitant increase in connectivity density. Since the rich club connections play a crucial role in network integration, the disruption of rich club connections might be related to the imbalance of integration and segregation of brain connectome. With a tendency of shifting towards a more segregated network, the efficiency of information processing across the brain might be weaken. And reductions in network efficiency were observed in MDD in many studies (17, 18). Taken together, childhood maltreatment may be linked with the dysconnectivity pattern among hub regions, alongside potential impairment in network integration. These findings highlight association between childhood maltreatment and the alterations of rich club organization in MDD.

In this study, we found that HC-CM group were presented with decreased connectivity strength and density in feeder connections, as well as elevated connectivity density in local connections, as compared to HC-nCM group. Additionally, the correlations between connectivity density in feeder connections and severity of childhood maltreatment were significantly negative in HCs, whereas in MDD these correlations showed positive. It seems that MDD and HCs exhibited different connection patterns in rich club organization when exposed to childhood maltreatment (41). In this study, childhood maltreatment was associated with reduced connectivity strength and density in rich club connections in patients with MDD. In contrast, within HCs, childhood maltreatment was related to decreased connectivity and density in feeder connections. These findings might suggest that distinct connection alterations could be presented in MDD patients and HCs when exposed to childhood maltreatment. As suggested in previous literature, the rich club regions are presented with a continuously high baseline activity and glucose metabolism, and could be particularly vulnerable to harmful mechanisms (24). This could be a possible interpretation for the reduction of rich club connection in MDD patients with childhood maltreatment exposure. However, in HCs, the individual with childhood maltreatment showed alterations in feeder connections rather than rich club connections. Here, we assumed that the disruption of feeder connections rather than rich club connections in HCs could be related to the influence of resilience. One plausible possibility is that resilient individuals could maintain rich club connections at the expense of disrupted feeder connection. As indicated by Teicher et al., there may be additional differences in the brains of the resilient subjects that enable these individuals to compensate for abnormalities in stress-susceptible structures (42). However, to the best of our knowledge, there have been no previous researches investigating the trade-off between rich club and feeder connections regarding the resilient individuals. Therefore, the interpretation of this assumption needed to be cautious, and further studies are warranted to validate it. Nonetheless, the alterations of feeder connections could be a critical neurological signature for resilience in the context of early life stress. Despite the distinct patterns of connection disruption, our studies demonstrated that childhood maltreatment was associated with widespread disruption of structural connectivity in MDD and HCs. As a chronic stress factor, childhood maltreatment could be related to the HPA axis dysregulation such as sensitization of the neuroendocrine stress response and increased central corticotropin-releasing factor activity (43). Based on previous literatures, stressed-related glucocorticoid changes are associated with anomalies in the function of oligodendrocytes, which could lead to structural changes that are reflected in myelin maintenance and plasticity (44). Moreover, the resilience is associated with adaptation in specific brain circuits such as threat and salience circuitry, and this adaptation could be involved in neurobiological pathways that are related to childhood maltreatment, which could be a plausible interpretation of distinct pattern of connection reduction in HCs (45). However, direct evidences supporting this hypothesis are still lacking, and longitudinal studies are needed to investigate the complicated effects of childhood maltreatment and resilience on rich club organization.

In this study, there were positive correlations between HAMD scores and connectivity strength in rich club and feeder connections, which appeared counterintuitive to the notion that connectivity disruption was associated with pathology. Here, we speculated that this finding could be related to a compensatory but maladaptive connectivity pattern. Specifically, we assumed that in MDD patients, the connections that didn’t sustain severe impairment might be presented with compensatory hyperconnectivity patterns. And hyperconnectivity in MDD have been reported in previous literatures (46, 47). However, the hyperconnectivity patterns might raise the metabolic burden to maintain brain network, which might accelerate the network dysfunction (48). Therefore, hyperconnectivity patterns may represent one potential neurobiological mechanism that is related to more severe symptoms. Moreover, this study identified distinct connectivity patterns in MDD and HC groups exposed to childhood maltreatment. These distinct patterns could have potential to serve as biomarkers for predicting MDD risk in individuals with a history of childhood maltreatment. However, longitudinal and prospective studies following a large cohort of individuals with a history of childhood maltreatment are warranted to validate their predictive values.

The identified rich club regions in current study included SFG, MFG, PreCG, PoCG, SMA, MOG, and MTG, which are involved in cognitive control, emotion regulation, somatosensory and motor, and visual processing (49–51). Notably, the same set of rich club regions was recognized across all groups. One possible interpretation is that the common set of rich club nodes is related to a special neuropathological condition in early stage. In early stage of disease, the hub nodes could be presented with pathological impairment and dysfunction, but they could maintain the hub status. With the progression of disease, the rich club nodes are impaired severely and couldn’t maintain the hub status, and then they could be presented with a complete loss of hub status. And then these groups might have different sets of rich club nodes. To validate our hypothesis, we further compared the nodal degree of rich club regions among these four groups. And the significant results in this validation analysis indicated that although these groups shared a common set of hub regions, they could be presented with intrinsic alterations in hub nodes due to pathological impairment. Moreover, longitudinal studies for monitoring the dynamic process of hub status fading are warranted to support this hypothesis.

Several limitations in the present study should be noted. First, the cross-sectional design of this study restricted our direct understanding about the effect of childhood maltreatment on the alterations of rich club organization in MDD. Longitudinal studies are needed in the future. Second, the childhood maltreatment was measured using a retrospective and self-reported questionnaire, and recall bias may lead to over- or underreported exposure of childhood maltreatment. Additionally, this questionnaire primarily focuses on domains of abuse and neglect. Other dimensions of childhood adversity, such as parental separation, school bullying, low-incomes, are not included in this assessment tool. The intrinsic defects in CTQ might partially contribute to modest correlations between childhood maltreatment and connectivity metrics. Third, some factors are known to influence the effect of childhood maltreatment, such as social support and resilience (52, 53). And the moderation of these factors could be another cause for the weak correlations in our study. However, our study didn’t explore the effects of these factors. Further studies investigating these psychosocial factors, as well as their interaction with childhood maltreatment, are warranted. Fourth, according to Mclaughlin et al.’s theory, the abuse and neglect are two specific dimensions of adversity (54, 55). In this model, abuse is referred to threat dimension, while neglect is involved in the dimension of deprivation. And these two maltreatment subtypes are associated with distinct neurological development. Notably, the findings of distinct mechanisms associated with different maltreatment subtypes are dependent on regional analysis, such as voxel-based analysis. However, the connectivity metrics of rich club organization in this study are global, and they are unable to capture the regional alterations that are related to different maltreatment subtypes. Fifth, this study only investigated the rich club organization for brain networks, other topological properties, such as small-worldness, global efficiency, clustering coefficient, and characteristic path length, may also be related to the neuropathological processes linking childhood maltreatment and MDD. And further studies on these metrics are warranted in future. Lastly, the sample size in our study may still be relatively small, and further researches with expanded sample size are warranted to verify the repeatability of our results.

## Conclusion

In summary, we explored the association between childhood maltreatment and alterations of rich club organization in structural brain connectome in first-episode, drug-naïve MDD. Our results suggested the disruption of rich club connections in MDD with childhood maltreatment. Furthermore, the MDD and HCs showed distinct connection patterns of rich club and non-rich club regions in detrimental influence of childhood maltreatment, which may indicate neuroimaging features associated with resilience to developing MDD in the context of early life stress.

## Data Availability

The raw data supporting the conclusions of this article will be made available by the authors, without undue reservation.
