# Supplementary material for: Disrupted rich club organization in structural brain networks is related to childhood maltreatment in major depressive disorder
Source: Front Psychiatry. 2026 Feb 26;17:1759133. doi: 10.3389/fpsyt.2026.1759133 (PMC12979451; doi:10.3389/fpsyt.2026.1759133)
Supplement: Supplementary file 1 [file Table1.docx]

**Table S1** Post-hoc comparisons of CTQ scores among MDD-CM, MDD-nCM, HC-CM, and HC-nCM groups.

| Comparison | CTQ | | EA | | PA | | SA | | EN | | PN | |
| --- | --- | --- | --- | --- | --- | --- | --- | --- | --- | --- | --- | --- |
|  | *diff* | *P_adj_* | *diff* | *P_adj_* | *diff* | *P_adj_* | *diff* | *P_adj_* | *diff* | *P_adj_* | *diff* | *P_adj_* |
| MDD-CM vs. MDD-nCM | 18.27 | < 0.001 | 3.92 | < 0.001 | 2.27 | < 0.001 | 1.59 | < 0.001 | 6.28 | < 0.001 | 4.34 | < 0.001 |
| MDD-CM vs. HC-CM | 13.44 | < 0.001 | 3.32 | < 0.001 | 1.03 | 0.173 | 0.74 | 0.309 | 5.28 | < 0.001 | 2.01 | < 0.001 |
| MDD-CM vs. HC-nCM | 24.87 | < 0.001 | 5.65 | < 0.001 | 2.52 | < 0.001 | 1.58 | < 0.001 | 8.63 | < 0.001 | 5.44 | < 0.001 |
| MDD-nCM vs. HC-CM | -4.83 | 0.104 | -0.60 | 0.846 | -1.24 | 0.119 | -0.85 | 0.279 | -0.99 | 0.606 | -2.33 | < 0.001 |
| MDD-nCM vs. HC-nCM | 6.60 | < 0.001 | 1.73 | 0.015 | 0.25 | 0.938 | -0.01 | 0.999 | 2.36 | 0.001 | 1.10 | 0.079 |
| HC-CM vs. HC-nCM | 11.43 | < 0.001 | 2.33 | 0.002 | 1.49 | 0.016 | 0.84 | 0.199 | 3.35 | < 0.001 | 3.43 | < 0.001 |

Abbreviations: MDD-CM, major depressive disorder with childhood maltreatment; MDD-nCM, major depressive disorder without childhood maltreatment; HC-CM, healthy controls with childhood maltreatment; HC-nCM, healthy controls without childhood maltreatment; CTQ, Childhood Trauma Questionnaire; EA, emotional abuse; PA, physical abuse; SA, sexual abuse; EN, emotional neglect; PN, physical neglect; *diff*, mean difference between groups; *P_adj_*, adjusted *P* value using Tukey’s honestly significant difference (HSD) method.
