# Supplementary material for: Disrupted rich club organization in structural brain networks is related to childhood maltreatment in major depressive disorder
Source: Front Psychiatry. 2026 Feb 26;17:1759133. doi: 10.3389/fpsyt.2026.1759133 (PMC12979451; doi:10.3389/fpsyt.2026.1759133)
Supplement: Supplementary file 2 [file Table2.docx]

**Table S2** Group comparisons of connectivity strength in rich club, feeder, and local connections among MDD-CM, MDD-nCM, HC-CM, and HC-nCM groups.

| Comparison | *diff* | *P* value | Corrected *P* value | Cohen’s d value |
| --- | --- | --- | --- | --- |
| Rich club connections |  |  |  |  |
| MDD-CM vs. MDD-nCM | -1.19e+03 | < 0.001 | < 0.001 | -0.714 |
| MDD-CM vs. HC-CM | -1.45e+03 | < 0.001 | < 0.001 | -0.915 |
| MDD-CM vs. HC-nCM | -9.27e+02 | < 0.001 | < 0.001 | -0.589 |
| MDD-nCM vs. HC-CM | -2.64e+02 | 0.533 | 0.533 | -0.148 |
| MDD-nCM vs. HC-nCM | 2.64e+02 | 0.395 | 0.474 | 0.157 |
| HC-CM vs. HC-nCM | 5.28e+02 | 0.117 | 0.175 | 0.328 |
| Feeder connections |  |  |  |  |
| MDD-CM vs. MDD-nCM | 5.33e+02 | 0.452 | 0.502 | 0.139 |
| MDD-CM vs. HC-CM | -5.32e+02 | 0.502 | 0.502 | -0.141 |
| MDD-CM vs. HC-nCM | -2.30e+03 | < 0.001 | < 0.001 | -0.615 |
| MDD-nCM vs. HC-CM | -1.06e+03 | 0.162 | 0.243 | -0.325 |
| MDD-nCM vs. HC-nCM | -2.83e+03 | < 0.001 | < 0.001 | -0.822 |
| HC-CM vs. HC-nCM | -1.76e+03 | 0.013 | 0.025 | -0.527 |
| Local connections |  |  |  |  |
| MDD-CM vs. MDD-nCM | -3.16e+03 | 0.040 | 0.080 | -0.378 |
| MDD-CM vs. HC-CM | -5.87e+03 | < 0.001 | 0.002 | -0.734 |
| MDD-CM vs. HC-nCM | -5.03e+03 | < 0.001 | < 0.001 | -0.603 |
| MDD-nCM vs. HC-CM | -2.71e+03 | 0.126 | 0.190 | -0.361 |
| MDD-nCM vs. HC-nCM | -1.87e+03 | 0.212 | 0.254 | -0.229 |
| HC-CM vs. HC-nCM | 8.39e+02 | 0.599 | 0.599 | 0.108 |

Abbreviations: MDD-CM, major depressive disorder with childhood maltreatment; MDD-nCM, major depressive disorder without childhood maltreatment; HC-CM, healthy controls with childhood maltreatment; HC-nCM, healthy controls without childhood maltreatment; *diff*, mean difference between groups, presented with scientific notation; Corrected *P* value, *P* value corrected using false discovery rate (FDR) method.
