# Supplementary material for: Disrupted rich club organization in structural brain networks is related to childhood maltreatment in major depressive disorder
Source: Front Psychiatry. 2026 Feb 26;17:1759133. doi: 10.3389/fpsyt.2026.1759133 (PMC12979451; doi:10.3389/fpsyt.2026.1759133)
Supplement: Supplementary file 3 [file Table3.docx]

**Table S3** Group comparisons of connectivity density in rich club, feeder, and local connections among MDD-CM, MDD-nCM, HC-CM, and HC-nCM groups.

| Comparison | *diff* | *P* value | Corrected *P* value | Cohen’s d value |
| --- | --- | --- | --- | --- |
| Rich club connections |  |  |  |  |
| MDD-CM vs. MDD-nCM | -8.72e-03 | 0.002 | 0.011 | -0.604 |
| MDD-CM vs. HC-CM | -7.37e-03 | 0.011 | 0.022 | -0.535 |
| MDD-CM vs. HC-nCM | -1.52e-03 | 0.463 | 0.556 | -0.113 |
| MDD-nCM vs. HC-CM | 1.35e-03 | 0.706 | 0.706 | 0.089 |
| MDD-nCM vs. HC-nCM | 7.20e-03 | 0.005 | 0.016 | 0.506 |
| HC-CM vs. HC-nCM | 5.85e-03 | 0.036 | 0.055 | 0.432 |
| Feeder connections |  |  |  |  |
| MDD-CM vs. MDD-nCM | 1.78e-02 | < 0.001 | < 0.001 | 1.215 |
| MDD-CM vs. HC-CM | 1.81e-02 | < 0.001 | < 0.001 | 1.221 |
| MDD-CM vs. HC-nCM | -2.23e-04 | 0.923 | 0.927 | -0.015 |
| MDD-nCM vs. HC-CM | 3.06e-04 | 0.927 | 0.927 | 0.021 |
| MDD-nCM vs. HC-nCM | -1.80e-02 | < 0.001 | < 0.001 | -1.181 |
| HC-CM vs. HC-nCM | -1.84e-02 | < 0.001 | < 0.001 | -1.184 |
| Local connections |  |  |  |  |
| MDD-CM vs. MDD-nCM | -9.10e-03 | 0.040 | 0.060 | -0.385 |
| MDD-CM vs. HC-CM | -1.08e-02 | 0.039 | 0.060 | -0.442 |
| MDD-CM vs. HC-nCM | 1.74e-03 | 0.626 | 0.751 | 0.075 |
| MDD-nCM vs. HC-CM | -1.65e-03 | 0.764 | 0.764 | -0.069 |
| MDD-nCM vs. HC-nCM | 1.08e-02 | 0.009 | 0.033 | 0.474 |
| HC-CM vs. HC-nCM | 1.25e-02 | 0.011 | 0.033 | 0.532 |

Abbreviations: MDD-CM, major depressive disorder with childhood maltreatment; MDD-nCM, major depressive disorder without childhood maltreatment; HC-CM, healthy controls with childhood maltreatment; HC-nCM, healthy controls without childhood maltreatment; *diff*, mean difference between groups, presented with scientific notation; Corrected *P* value, *P* value corrected using false discovery rate (FDR) method.
