# Supplementary material for: Disrupted rich club organization in structural brain networks is related to childhood maltreatment in major depressive disorder
Source: Front Psychiatry. 2026 Feb 26;17:1759133. doi: 10.3389/fpsyt.2026.1759133 (PMC12979451; doi:10.3389/fpsyt.2026.1759133)
Supplement: Supplementary file 4 [file Table4.docx]

**Table S4** Group comparisons of nodal degree in bilateral SFG, right PreCG, right MTG, and right MFG among MDD-CM, MDD-nCM, HC-CM, and HC-nCM groups.

| Comparison | *diff* | *P* value | Corrected *P* value | Cohen’s d value |
| --- | --- | --- | --- | --- |
| L-SFG |  |  |  |  |
| MDD-CM vs. MDD-nCM | -0.75 | 0.519 | 0.779 | -0.123 |
| MDD-CM vs. HC-CM | -0.54 | 0.698 | 0.837 | -0.084 |
| MDD-CM vs. HC-nCM | -2.23 | 0.007 | 0.040 | -0.403 |
| MDD-nCM vs. HC-CM | 0.21 | 0.872 | 0.872 | 0.041 |
| MDD-nCM vs. HC-nCM | -1.48 | 0.063 | 0.149 | -0.343 |
| HC-CM vs. HC-nCM | -1.69 | 0.075 | 0.149 | -0.372 |
| R-SFG |  |  |  |  |
| MDD-CM vs. MDD-nCM | -1.55 | 0.214 | 0.320 | -0.235 |
| MDD-CM vs. HC-CM | -2.56 | 0.060 | 0.162 | -0.394 |
| MDD-CM vs. HC-nCM | -3.14 | 0.001 | 0.006 | -0.520 |
| MDD-nCM vs. HC-CM | -1.00 | 0.392 | 0.471 | -0.205 |
| MDD-nCM vs. HC-nCM | -1.59 | 0.081 | 0.162 | -0.319 |
| HC-CM vs. HC-nCM | -0.58 | 0.546 | 0.546 | -0.127 |
| R-PreCG |  |  |  |  |
| MDD-CM vs. MDD-nCM | -1.23 | 0.182 | 0.285 | -0.250 |
| MDD-CM vs. HC-CM | -1.97 | 0.051 | 0.152 | -0.410 |
| MDD-CM vs. HC-nCM | -2.12 | 0.001 | 0.008 | -0.483 |
| MDD-nCM vs. HC-CM | -0.74 | 0.437 | 0.525 | -0.188 |
| MDD-nCM vs. HC-nCM | -0.89 | 0.190 | 0.285 | -0.240 |
| HC-CM vs. HC-nCM | -0.15 | 0.833 | 0.833 | -0.044 |
| R-MTG |  |  |  |  |
| MDD-CM vs. MDD-nCM | 0.25 | 0.885 | 0.885 | 0.028 |
| MDD-CM vs. HC-CM | -1.49 | 0.387 | 0.465 | -0.184 |
| MDD-CM vs. HC-nCM | -3.01 | 0.009 | 0.040 | -0.392 |
| MDD-nCM vs. HC-CM | -1.74 | 0.321 | 0.465 | -0.233 |
| MDD-nCM vs. HC-nCM | -3.26 | 0.013 | 0.040 | -0.455 |
| HC-CM vs. HC-nCM | -1.51 | 0.240 | 0.465 | -0.247 |
| R-MFG |  |  |  |  |
| MDD-CM vs. MDD-nCM | -0.10 | 0.941 | 0.941 | -0.015 |
| MDD-CM vs. HC-CM | 0.56 | 0.690 | 0.828 | 0.087 |
| MDD-CM vs. HC-nCM | -2.50 | 0.001 | 0.002 | -0.487 |
| MDD-nCM vs. HC-CM | 0.66 | 0.663 | 0.828 | 0.106 |
| MDD-nCM vs. HC-nCM | -2.40 | 0.002 | 0.005 | -0.520 |
| HC-CM vs. HC-nCM | -3.06 | < 0.001 | 0.002 | -0.800 |

Abbreviations: L, left; R, right; SFG, superior frontal gyrus; PreCG, precentral gyrus; MTG, middle temporal gyrus; MFG, middle frontal gyrus; MDD-CM, major depressive disorder with childhood maltreatment; MDD-nCM, major depressive disorder without childhood maltreatment; HC-CM, healthy controls with childhood maltreatment; HC-nCM, healthy controls without childhood maltreatment; *diff*, mean difference between groups; Corrected *P* value, *P* value corrected using false discovery rate (FDR) method.
